# Radiological dose reconstruction for birds reconciles outcomes of Fukushima with knowledge of dose-effect relationships

**DOI:** 10.1038/srep16594

**Published:** 2015-11-16

**Authors:** Jacqueline Garnier-Laplace, Karine Beaugelin-Seiller, Claire Della-Vedova, Jean-Michel Métivier, Christian Ritz, Timothy A. Mousseau, Anders Pape Møller

**Affiliations:** 1 Institut de Radioprotection et de Sûreté Nucléaire, Pôle Radioprotection, Environnement, Déchets, Crise, PRP-ENV/SERIS, Cadarache, Bâtiment 159, BP3, F-13115 Saint Paul lez Durance Cedex, France; 2 Department of Nutrition, Exercise and Sports Faculty of Science, University of Copenhagen, Rolighedsvej 26 DK-1958 Frederiksberg C, Denmark; 3 Department of Biological Sciences, University of South Carolina, Columbia, SC 29208, USA; 4 Laboratoire d’Ecologie, Systématique et Evolution, CNRS UMR 8079, Université Paris-Sud, Bâtiment 362, F-91405 Orsay Cedex, France

## Abstract

We reconstructed the radiological dose for birds observed at 300 census sites in the 50-km northwest area affected by the accident at the Fukushima Daiichi nuclear power plant over 2011–2014. Substituting the ambient dose rate measured at the census points (from 0.16 to 31 μGy h^−1^) with the dose rate reconstructed for adult birds of each species (from 0.3 to 97 μGy h^−1^), we confirmed that the overall bird abundance at Fukushima decreased with increasing total doses. This relationship was directly consistent with exposure levels found in the literature to induce physiological disturbances in birds. Among the 57 species constituting the observed bird community, we found that 90% were likely chronically exposed at a dose rate that could potentially affect their reproductive success. We quantified a loss of 22.6% of the total number of individuals per increment of one unit log_10_-tansformed total dose (in Gy), over the four-year post-accident period in the explored area. We estimated that a total dose of 0.55 Gy reduced by 50% the total number of birds in the study area over 2011–2014. The data also suggest a significant positive relationship between total dose and species diversity.

The nuclear disaster at the Fukushima Daiichi nuclear power plant (NPP) following the massive earthquake and devastating tsunami on 11 March 2011 was rated at 7 which is the highest level on the international nuclear disaster scale, putting it on par with the accident at Chernobyl. Vast amounts of radioactive materials were released, estimated as ca. 10–15% (i.e. 400–630 PBq) and 35% (i.e. 58 PBq) of the total iodine and cesium radioisotopes emitted by the Chernobyl accident, respectively^1^,^2^. The effect of these radioactive releases on abundance, condition and rate of reproduction in birds and other animals has been the subject of investigation during 2011–2014 within the 50-km area northwest of Fukushima Daiichi NPP^3^,^4^,^5^,^6^,^7^. In total, 400 sampling points (in 2011, 300 points) in areas within and outside the 20-km exclusion zone were visited annually to observe abundance and diversity of birds and other organisms, as well as their reproductive state; a number of environmental variables including ambient radiation level at the exact census points were also recorded. In total this database contains information for 1500 point censuses (300 identical points from 2011 to 2014 with an additional 100 more points from 2012 to 2014).

Different analyses from this dataset have been published, showing reduced species richness and abundance in areas of relatively high ambient radiation level^7^,^8^, as also shown for the Chernobyl Exclusion Zone – CEZ^4^,^9^,^10^,^11^,^12^. These analyses were performed taking into account a number of potentially confounding environmental variables such as descriptors of the census conditions (e.g., time, cloud cover, wind, temperature) and of landscape (i.e. cover type such as e.g., farmland, grass, coniferous trees)^4^,^6^,^7^,^9^,^10^,^11^,^12^. Analyses based on Generalized Linear Mixed Models (GLMM) with Poisson distributed data, least squares analyses and non-parametric Spearman rank order correlations showed similar conclusions^6^. Thus the conclusions appeared to be independent of statistical analyses. The most recent analyses of the entire dataset collected in the Fukushima area by Møller *et al.*^6^ showed the abundance of birds decreased with increasing level of ambient radiation, with significant variation among species, and the relationship between abundance and radiation became increasingly negative over time.

Among the 154 bird species present in the inventory at Chernobyl and Fukushima, species with small body size and hence relatively high food consumption rates were more negatively impacted by radiation at Fukushima and Chernobyl^7^. Secondary consumers showed stronger negative effects of radiation on abundance than herbivores, and more so at Fukushima than at Chernobyl^7^. There was no overall effect of migration behavior, but migratory species were more negatively impacted in Chernobyl, while residents were more negatively impacted in Fukushima^7^ perhaps reflecting the effects of high acute exposures to resident species during the initial phase of the disaster. Species with carotenoid and pheomelanin plumage pigments associated with antioxidant status showed stronger negative effects, especially in Chernobyl compared to Fukushima, while species with eumelanic coloration, which is not related to antioxidant status, did not show such an effect. These differences between Chernobyl and Fukushima may reflect differences in duration of exposure, differences in radioactive isotopes still being present in the radioactive deposits at the census periods, and differences in potential accumulation of mutations since sampled individuals are offspring from a much smaller number of generations in Fukushima than in Chernobyl^7^,^13^.

Recently, Garnier-Laplace *et al.*^14^ proposed a re-assessment of inter-species radio sensitivity emphasizing that organisms in their natural environment from the CEZ appeared to be ca. eight times more sensitive to radiation than they were under laboratory conditions. This is not surprising given that organisms under field conditions may suffer from poor nutrition and/or unfavorable abiotic factors, and parasitism and predation with strong effects on abundance and species diversity, while lab populations are usually kept under optimal conditions, partly as a consequence of legislation related to research ethics. Additionally the duration of exposure is clearly a major feature that distinguishes laboratory and field experiments, since for the same exposure dose rate, the resulting absorbed doses are drastically different with ultimately much higher doses in the field than in the laboratory. Finally, any shift in sensitivity between field and laboratory may be due to the presence of other stressors such as stable contaminants and/or to selection of strains through phenotypic acclimation, DNA methylation, genetic adaptation across generations, or multigenerational phenotypic effects (e.g., maternal effects)^15^,^16^,^17^.

On the basis of ecological data describing bird diversity and abundance in the 50-km contaminated area around Fukushima published by Møller *et al.*^6^,^7^, the objectives of the present study were to reconstruct the total dose (and dose rates) of individual birds and to analyse whether this new approach might better explain the intensity and type of observed effects on bird abundance and species diversity. More precisely, we reassessed the entire dataset by substituting the ambient radiation level previously used by Møller *et al.*^6^,^7^ by the total doses reconstructed for birds. As noticed by Beresford *et al.*^18^,^19^, the ambient radiation level is assumed to be a biased radiation dose indicator, especially when several species of free-ranging animals may be impacted. For a given contaminated environment, where ambient radiation level is constant, radiological dose rate absorbed by living organisms may vary by several orders of magnitude since animal biodiversity is characterized by a variety of species-specific morphological and ecological features (e.g., types of habitats, diet) that may greatly influence the range of absorbed dose. Any dose (rate) reconstruction needs to assess its two elementary components: the external dose (rate) from the radio-contaminated habitat and the internal dose (rate) from internalization of radionuclides. In this study, both components were assessed on the basis of (i) the stylized morphological and ecological features of the species and (ii) the radioactive concentration of the soil using GPS coordinates from each census point combined with the radioactive contamination maps of the Fukushima area, the latter being based on field monitoring in soils performed by Japanese university scientists, from June-July 2011^20^. We re-analysed the ecological dataset with reconstructed total dose for bird individuals (sum of internal dose and external dose) at census points to investigate its relationship with temporal and spatial records of bird abundance and species richness. We also examined the relationship between absorbed dose and the abundance and diversity of birds and we compared estimated critical radiotoxicity endpoints (e.g., dose giving 50% effects on the abundance) with various radiation effect benchmarks published by international organisations.

## Results

### Comparison between ambient radiation levels and estimated bird dose rates and total doses

**Figure 1:**
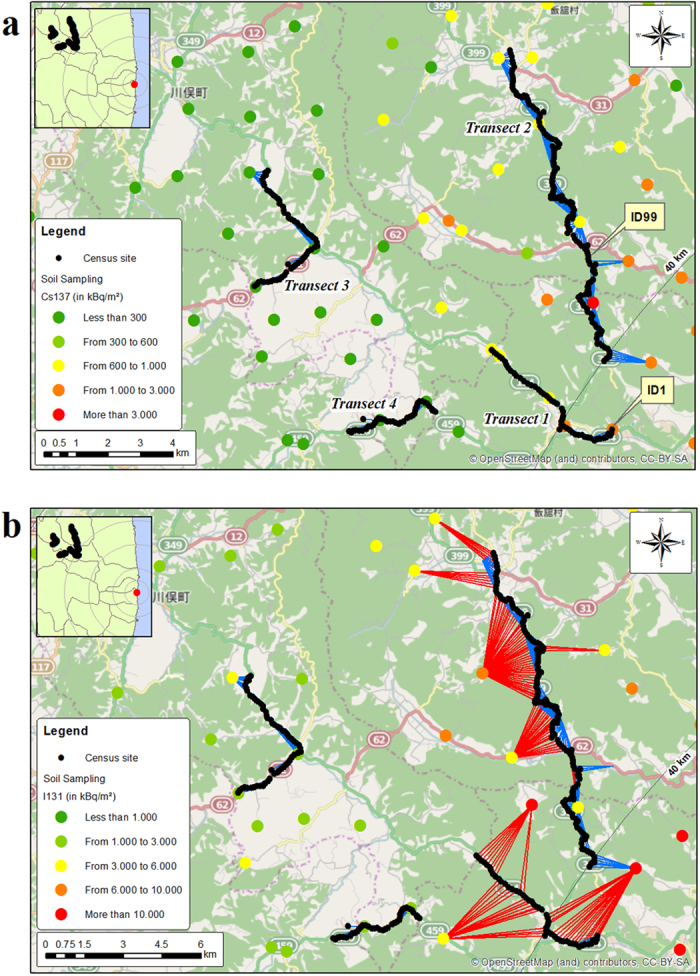
Location of the 300 census sites for birds distributed along four transects. Each census site was associated with soil activity ((**a**) ^137^Cs equal to ^134^Cs and (**b**) ^131^I) equal to that measured at the nearest soil sampling point. Note that several neighbor census sites were associated with the same measurement point. Maps were created with GIS software (ArcGIS 10.1) http://support.esri.com/en/downloads/patches-servicepacks/view/productid/66/metaid/2064 with geographical background using data available under the Open Database Licence (“© OpenStreetMap and contributors”; cartography licensed as CC BY-SA) http://www.openstreetmap.org/copyright.

**Figure 2:**
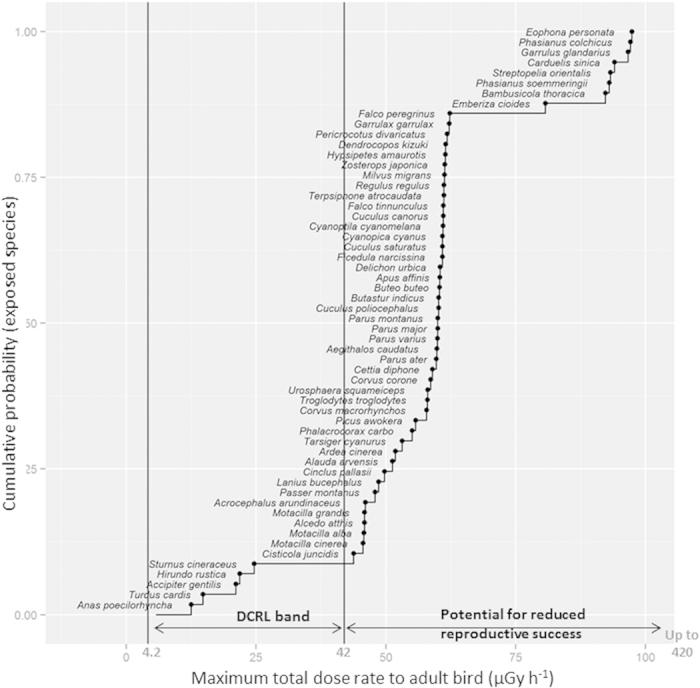
Empirical cumulative probability distribution of maximum estimated dose rates for the 57 species, 300 sites and four census years. The adult bird exposure levels were compared to ranges of biological effects reported by ICRP^21^.

The ratio between the reconstructed absorbed dose rates for adults of all bird species and the measured ambient radiation levels according to site, year and species, varied from 0.1 to 20. For example, the minimum value for this ratio was obtained in 2011 for *Anas poecilorhyncha* at site ID99 (Fig. 1a), where the ambient level was measured at 19 μGy h^−1^ and the maximum ratio (x20) was obtained in 2014 for site ID1 (Fig. 1a) and *Phasianus colchicus.* Table 1 reports the ranges of variation in ambient radiation levels among sites per year and the way that this heterogeneity is reflected in the calculated dose rates among species. Globally, whatever year, where ambient dose rate varied by two orders of magnitude among sites, variation in absorbed dose rates among species and among sites also varied by two orders of magnitude, the minimum value being estimated for *Anas poecilorhyncha* and the maximum for *Phasianus colchicus* and *Eophona personata.* Additionally, for a given site, the absorbed dose rate varied eight-fold among the 57 species mainly due to variation in Concentration Ratio values selected for Cs isotopes (supplementary Table S4). For a given species, the absorbed dose rate varied by a factor of 44 among the 300 sites. The empirical distribution of the maximum values per species obtained for the full dataset of the reconstructed dose rates for adult birds is given in Fig. 2.

### Testing the relationships between bird abundance and Simpson’s index of diversity, respectively, and total doses

When averaged for the full dataset (i.e. 57 species x 300 sites x 4-yr), the estimated total dose was 0.13 Gy (range: 0.0031–1.23 Gy; N = 1190). The average observed total number of birds per site was 6.17 (range: 0–49; N = 1200). The average observed Simpson’s Index of diversity was 0.66 (range: 0–1; N = 1200).

**Figure 3:**
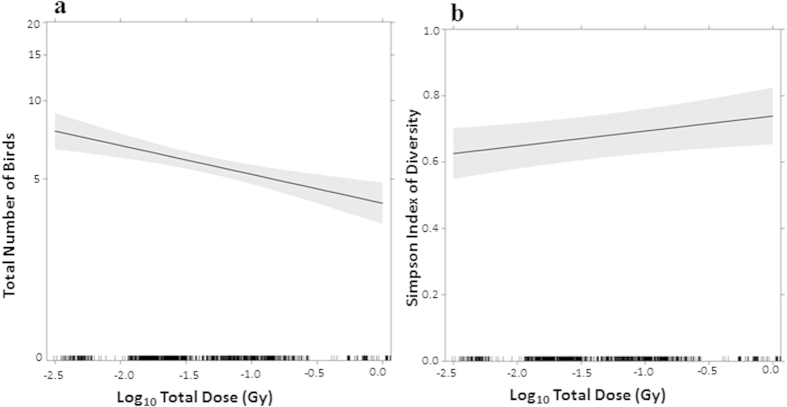
(**a**) Effect display^44^ for the calculated total dose (log_10_-transformed) predictor of total number of birds (TNB), in the fitted GLMM. After controlling for the possible confounding variables, the total number of birds decreased by 22.6% per unit of log_10_ total dose. Note that the vertical axis is on a scale of the linear predictor with axis tick marks labelled on the scale of the response (TNB). The decrease is highly significant (z = **−**4.06, p < 0.0001; unstandardised partial slope (SE) = **−**0.256 (0.063)). (**b**) Effect display for the calculated total dose (log_10_-transformed) predictor of Simpson’s index of diversity, in the fitted LMM. After controlling for the possible confounding variables, the probability that two individuals belonged to different species increases linearly by 4.5% per unit of log_10_ total dose. The increase is significant (t = 2.3, p < 0.025; unstandardised partial slope (SE) = **−**0.045 (0.020)); Grey bands in the panels represent the 95% confidence intervals around the fits.

The total number of birds decreased significantly with increasing total dose (log_10_-transformed) (Table 2; z = −4.060, p < 0.0001; standardised partial slope (SE) = −0.299 (0.074)), altitude (z = −5.980, p < 0.0001; standardised partial slope (SE) = −0.275 (0.046)), grass as land cover (z = −4.980, p < 0.0001; standardised partial slope (SE) = −0.173 (0.035)) and temperature (z = −2.400, p = 0.016; standardised partial slope (SE) = −0.182 (0.076)). The total number of birds increased significantly with increasing “cover by farmland” (z = 5.790, p < 0.0001; standardised partial slope (SE) = 0.266 (0.046)). In addition, the total number of birds differed significantly among the categories of wind speed, and had a significant curvilinear relation with time (z = 5.050, p < 0.0001; standardised coefficient (SE) = 0.352 (0.070)). Total dose (log_10_-transformed) was among predictors having the greatest effects on the total number of birds. Using the unstandardised partial slope of total dose (log_10_-transformed) (Table 2), we estimated a loss of 22.6% with CI 95% [12.4; 31.6] (i.e. the loss being calculated as [exp (−0.256) − 1]) of the total number of birds per unit of log_10_-transformed total dose (in Gy), i.e. each time the total dose increases by a factor 10 (Fig. 3a).

Species diversity expressed as the Simpson’s index of diversity increased significantly with increasing total dose (log_10_-transformed) (Table 3; t = 2.302, p = 0.025 ; partial standardized slope (SE) = 0.053 (0.02)) and with “cover by farmland” (t = 6.594, p < 0.0001; partial standardised slope (SE) = 0.089 (0.014)) and decreased with increasing time (t = −5.643, p < 0.0001; partial standardised slope (SE) = −0.084 (0.015)) and increasing altitude (t = −2.888, p = 0.004; partial standardised slope (SE) = −0.040 (0.014)). In addition, bird species diversity differed significantly among wind speed categories. After controlling for all the other predictors, the probability of observing two individuals of different species increased linearly by ca. 4.5% with CI 95% [0.6; 8.4] (unstandardised coefficient) per unit of log_10_-transformed total dose (in Gy) (Fig. 3b).

### Further investigating the relationship between bird abundance and total dose

**Figure 4:**
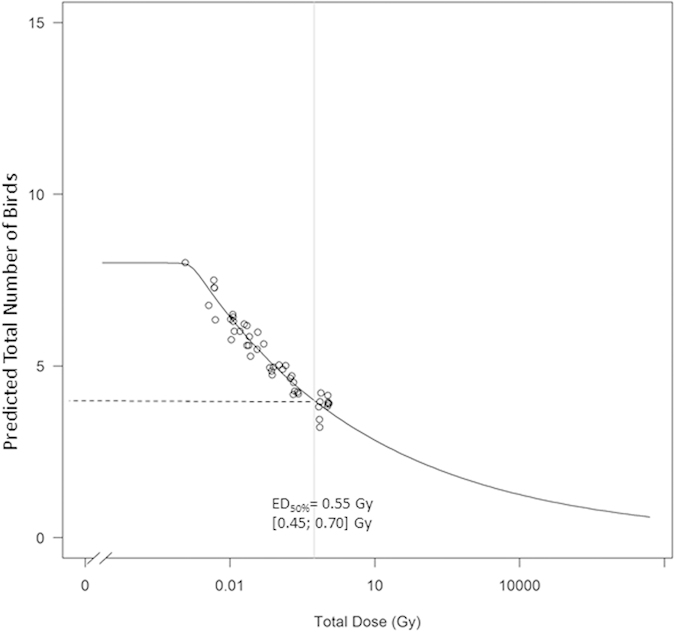
Log logistic model fitted to randomly predicted total number of birds derived from the global GLMM, and its ED_50_ prediction and associated 95% Confidence Interval (ED_50_ is the total absorbed dose causing a 50% reduction in the total number of birds).

Dose –response modelling based on a subset of randomly selected total doses and corresponding total number of birds estimated from the previously fitted GLMM (Table 2) allowed us to predict that a total absorbed dose of 0.55 Gy with CI 95% [0.45; 0.70] caused a 50% loss in the total number of birds (Fig. 4).

## Discussion

In this study, substituting the ambient dose rate measured at the census point (from 0.16 to 31 μGy h^−1^) by the transformed dose variable using radiological dose rate reconstructed for adult birds of each species (from 0.3 to 97 μGy h^−1^), we confirmed that the overall abundance of birds at Fukushima during 2011–2014 decreased with increasing absorbed doses (Fig. 3a). As such, this relationship is directly consistent with exposure levels cited in the literature that are known to induce physiological disturbances in terrestrial vertebrates or more specifically in birds. The International Committee for Radiological Protection recommended in its Publication 108^21^ (which is dedicated to concepts and approach for radiological protection of the environment) to use Derived Consideration Reference Levels (DCRL) values corresponding to the “*band of dose rates within which there is likely to be some chance of deleterious effects occurring to individuals of such type of organism”*. For birds, the DCRL range is 0.1–1 mGy d^−1^ (or 4–42 μGy h^−1^). On the basis of an expert judgement from a critical literature review, ICRP^21^ also reported the dose rate ranges where various significant effects were described in birds: mortality in adults (>1000 mGy d^−1^ or 42,000 μGy h^−1^), long-term effects on developing embryos (e.g. reduction in life-span) (100–1000 mGy d^−1^ or 4,200 to 42,000 μGy h^−1^), increased morbidity (10–100 or 420 to 4,200 μGy h^−1^), and reduced reproductive success (1–10 mGy d^−1^ or 42 to 420 μGy h^−1^). We considered the empirical cumulative distribution of the maximum exposure levels per species across the 300 sites and the four years, along with the assumption that radio sensitivity is similar among bird species. Comparing with ICRP bands previously described, we found that 90% of the 57 species constituting the observed bird community were likely chronically exposed at a dose rate that could potentially affect their reproductive success (Fig. 2). This finding is valid when reasoning at the bird community level in the studied area, but needs to be refined if we want to rank species at risk since the most exposed species is not necessarily the most radiosensitive.

More precisely, we were able to quantify a loss of 22.6% of the total number of individuals per increment of one unit log_10_ in the total dose over the four-year post-accident period in the study area. On the basis of the present dataset and the global GLMM we implemented, we were able to predict a value of 0.55 Gy for the total dose inducing 50% loss in the total number of birds at a census site (ED_50_) (Fig. 4). This value can be compared to the transitional vertebrate Predicted No Effect Dose Rate (PNEDR) proposed by Garnier-Laplace *et al.*^22^ for chronic exposure to ionising radiation which equals 2 μGy h^−1^. This benchmark, being derived from a minimalist dataset, was considered as indicative of the order of magnitude only, rather than being a definitive number. When multiplied by the exposure duration of birds in this study, this benchmark can be converted into a range which extends from 5.4 mGy in 2011 to 58 mGy in 2014. This range is consistently 10 to 100 times lower than the estimated ED_50_ of 0.55 Gy. Since the Simpson’s biodiversity index analysis was more quantitative than qualitative, it remains difficult to identify whether this loss in number of individuals was peculiar for a specific species.

We demonstrated that the relationship between total doses and species diversity, estimated as Simpson’s index, increased significantly with total doses (Fig. 3b). One could speculate that an increase in diversity may result indirectly from a decrease in abundance, as a result of homogenization of abundances for various species. Møller *et al.*^6^ concluded that in the Fukushima area, although bird species on average declined in abundance with increasing ambient levels, several species increased in abundance due to changes in land-use and/or to competitive release. From ecological surveys implemented in the Chernobyl Exclusion Zone^4^,^9^,^10^ in sites covering ambient radiation levels ranging from 0.01 to 4500 μGy h^−1^, statistical analyses provided evidence of dramatic effects on species richness and abundance of “forest” birds and skewed age and sex ratios in many birds populations. It would be informative to reassess the data collected in previous years concerning effects on the biota of Chernobyl Exclusion Zone using the same approach we have implemented in the present study to reconstruct in an ecologically relevant way the radiological dose to biota. As demonstrated for this study in the Fukushima area, our method potentially enhances the relevance and consistency of the analysis of causal relationship between levels of exposure (dose or dose rate) and type and intensity of observed effects. Other studies published after the Fukushima accident, such as those dedicated to the pale blue grass butterfly^23^,^24^, earthworms^25^ and wild macaques^26^, might also benefit from a re-analysis that includes a rigorous dose assessment.

Importantly, the method presented here to obtain relatively refined estimates of bird individual dose, have permitted a much finer-scale analysis of the factors shaping the biological endpoints of interest, in this case, species level performance within the context of the broader bird community exposed to radioactive substances accidentally released into the environment. However, like any modeling approach, our dose estimates were based on assumptions and models, and present therefore limitations and uncertainties.

One among them is the use of real measured soil activity concentrations at the closest soil sampling stations to any bird census point. Such input data used to reconstruct the bird dose takes into account roughly the spatial heterogeneity of radioactive deposits and avoids more complex and uncertain spatial interpolation. Moreover, bird dose reconstruction ideally needs to be combined with the spatial home range for individuals of the 57 species. This home range is generally known to vary depending on habitat type/quality, population density, behavior, physiology, etc.^27^. To our knowledge meaningful data for species home ranges for individuals only exist for a few of the 57 species of interest in this study. So our approach seems a reasonable compromise to address such lack of knowledge. Additionally, we calculated a maximum cumulated dose per species since we adopted the hypothesis that all species were present at the time of the accident and that dose accumulation was not limited by life span. Life span per species is difficult to estimate robustly since it can be influenced by environmental conditions. We considered that cumulative dose over a 4-yr period for the maximum was still quite realistic for the 57 species. Analysis of longer term data will require an additional assumption to limit the accumulated dose in a realistic manner with regard to species’ life spans.

Another important source of uncertainty for dose estimates is that we ignored the early life stages for dose reconstruction in adult birds. It would have been highly speculative to calculate the dose absorbed by eggs and nestlings although eggs and nestlings are known to be particularly radiosensitive compared to adults. This may have particular relevance here given that the period of highest exposures overlapped with the breeding season for many species. For any given species, the dose assessment for eggs and nestlings requires data on nest material, duration of incubation and rearing duration of chicks in the nest. Such information varies greatly across species and may have been modified over the four-year census period since they are sensitive to changes in environmental factors. To give the order of magnitude of the uncertainty introduced by voluntary omission of early life stages in bird dose assessment, we can refer to a recent field study performed in May-June 2012 by Bonisoli-Alquati *et al.*^3^ dedicated to nestling barn swallows *Hirundo rustica* in Fukushima 80 km NW of the contaminated area. The authors measured external dose (rate) of nestlings by using thermoluminescent dosimeters left in the nest and found that it varied from 0.15 to 4.9 mGy (0.23 to 7.52 μGy h^−1^). Nest materials contributed dominantly to the dose of nestlings. This value represents ca. 50% of the dose rate we calculated for adult barn swallows in 2012, the latter varying from 0.4 to 18 μGy h^−1^ across the 300 census sites. However, when converted into total dose by multiplying by the duration over which these dose rates apply, the contribution represents less than 5% of the total dose, which is the basic variable used in our statistical analysis. Adult barn swallows considered in our study in 2012 had absorbed from 4.8 to 210 mGy since the accident, depending on the site at which they were observed. Although this contribution of early stage dose to total dose may vary across the 57 species of this study, we can reasonably assume that it is of minor importance. Moreover this effect is balanced by our conservative choice to calculate the total dose only for birds present at the moment of the accident. Finally, it seems reasonable to assume that any such effects will add noise to the data and not result in systematic bias in the conclusions.

Another limitation to our present dose calculation is the use of the simple Concentration Ratio (CR) concept to model the internal dose of adult birds. This commonly used approach generally leads to a range of CR values spanning several orders of magnitude across species of birds. For example for cesium, IAEA^28^ reported a range of variation from 0.0014 to 16 (Bq kg^−1^ fresh wet: Bq kg^−1^, dry weight soil) on the basis of a dataset of 180 observations. In the present study where we followed recommendations from IAEA^28^ and Brown *et al.*^29^ for filling data gaps, we were able to reduce the uncertainty by selecting fit-for-purpose data per species, i.e. using species-specific CR values when possible or adopting taxonomically based extrapolation rules for combinations (species, radionuclide) where data were lacking. These rules consist of selecting a CR value acquired first for a similar genus, then for another species with similar diet and lastly among vertebrate classes such as mammals considered of “similar taxonomy”. We selected the CR method for our analysis since our dose estimates mainly dealt with the late phase of the Fukushima accident, over 4 years since July 2011, when soil radionuclide concentrations were not subject to rapid changes over time. We recognize however that it is much less appropriate for the 2011 dose estimation due to the rapid changes of soil concentrations of short-lived radioisotopes in the early phase after the accident. According to the United Nations Science Committee on the Effects of Atomic Radiation (UNSCEAR)^2^, these short-lived radioisotopes contributed to high dose rates, but of very short duration (typically a few days) due to rapid radioactive decay. For 2011, uncertainties that would have been introduced by the use of dynamic models would have been much larger since this type of models requires more parameters.

Although assumptions are needed to properly perform dose calculations as discussed above, reconstructed doses as proposed in this paper are likely the most appropriate variable reflecting the intensity of the radiological exposure. Dose reconstruction allows for accounting of differences in morphology and ecology among species in a comparable manner, as well as spatial heterogeneity in soil radioactive contamination. Our estimates of absorbed doses for adult birds are in agreement with these previous publications. Garnier-Laplace *et al.*^30^ calculated dose rates of birds for the first 3-week period after the Fukushima accident and reported a value of 1.5 mGy d^−1^ (63 μGy h^−1^) accounting for ^134^Cs, ^137^Cs and ^131^I and a series of short-lived radioisotopes. This value fits very well within the bounds of the inter-species range calculated for the present study in 2011 (0.3 to 97 μGy h^−1^). UNSCEAR^2^ also assessed the dose rate absorbed by terrestrial birds during the first two months after the accident. Based largely on measured data, the Committee estimated the 95^th^ percentile of the dose rate distribution in the area of Koriyama City (50 to 100 km west of the Fukushima Daiichi site) in June 2011 at 1.5 μGy h^−1^. Using a similar methodology as the one used here, the Committee also estimated a dose rate of 21 μGy h^−1^ for duck in June 2011 in Okuma town, an area with relatively high deposition density inside the Fukushima exclusion zone.

This paper encourages performing ad hoc statistical data treatment of field datasets where ambient dose levels are substituted by radiological dose (rates) to biota of interest. This would help advance the understanding of radiation effect mechanisms and their relation with exposure modalities such as acute vs. chronic exposure to various radiation types, along with realistic ecological conditions in the field where a number of environmental factors are to be taken into account. Additionally, it is of major importance to collect information on dose-effect relationships acquired in radioactively contaminated area for ecologically relevant endpoints, i.e. those with potential consequences in terms of ecosystem structure and function and population demography. Such knowledge will help to elucidate findings from the first comparison of species’ radiosensitivity between controlled laboratory conditions and natural environments performed by Garnier-Laplace *et al.*^14^, where the meta-analysis failed to discriminate among the various reasons that could drive the apparent higher radio sensitivity of species in the field. For instance, field exposure-effect relationships can be modified due to the combination of radiotoxicity effects on growth rate/reproduction and of genetic diversity, competition, predation and abiotic factors including other pollutants in addition to radionuclides. This point was also recently highlighted in Bonisoli-Alquati *et al.*^3^, along with the issue of challenging effect benchmarks published by a number of international organisations to be used as references for ionising radiation in ecological risk assessments.

Inclusion of information concerning the duration of exposure to ionising radiation by migrants and residents would extend the present study. The absence of such information is likely to result in conservative conclusions in the present study. Likewise CR data were derived from studies varying extensively in sampling effort from a single to numerous individuals. Such differences in sampling effort must be addressed because they are known to strongly bias statistical analyses of comparative data^31^. Finally, the findings of our present study are clearly consistent with the present knowledge of dose-effect relationship for ionising radiation. It reinforces the need for continuing ecological surveys in the Fukushima affected area, but also at Chernobyl and other sites exposed to ionising radiation. The merger of advanced ecological methods with sophisticated dose reconstruction models presented in this paper has never been attempted before and is promising for improving the understanding of ecological consequences of chronic exposure to ionising radiation.

## Methods

### Study sites and bird census methods

We re-analysed a data subset described in Møller *et al.*^6^. Briefly, it reports breeding bird censuses at 300 sampling sites visited once a year at the beginning of July 2011–2014. To avoid giving less weight to the year 2011 in the present reassessment based on a global data analysis (300 census points x 4 years), we did not use the 100 sites that were added to the original study from 2012 to 2014. Each sampling site was localized using GPS coordinates and altitude. The 300 census sites were spaced by ca. 100 m and are for logistic and legal reasons distributed along four transects (Fig. 1). The dataset reports specific abundances for 57 species of birds using the point count census method. The repeatability and reliability of this approach has been tested in previous studies in Chernobyl and elsewhere^32^,^33^, but also in numerous other studies since the initial study by Blondel *et al.*^34^. Various environmental descriptors collected per site by Møller *et al.*^6^ were also used as potential covariates in the present analysis with the same justification as in the original study. This list is as follows: the time of day at the start of counts at each point, the time squared to reflect the curvilinear relation between bird behavioral activity and time of day^33^, cloud cover, temperature, wind force, and ground coverage 50 m around the census point to represent the type of habitats (as % of grass, bush, tree, deciduous forest, farmland (i.e. arable land), coniferous forest cover, recorded to the nearest 10%). In addition, radiation was measured at ground level at each survey point after having conducted the survey (thus making the survey blindly with respect to radiation level) using a hand-held dosimeter (Model: Inspector, International Medcom, Inc, Sebastopol, CA, USA). We measured levels several (2–3) times at each site one measurement right after another and averaged the measurements (see Møller *et al.*^6^ for detailed methods).

### Basic lines for reconstruction of total radiological doses absorbed by different bird species

The biological effects of ionising radiation are generally dependent on the dose absorbed, which is linked to the energy deposited in the body of living organisms from two pathways. One pathway is external irradiation from the surrounding contamination such as e.g. from contaminated soils, mainly due to gamma rays, but also possibly to beta radiation for small organisms (of sizes <1 cm). A second pathway is internal irradiation due to internalization of radionuclides, whatever the physiological process involved. The radiation dose for an organism is the total quantity of energy absorbed from ionising radiation per unit mass of tissue (1 Gy = 1 J kg^−1^ of tissue), and the dose rate refers to the energy absorbed over time (e.g., μGy h^−1^). Radioactive decay results in different types of emissions that vary in energy among different radionuclides, and the effectiveness of radiation in causing biological damage is related to the type of radiation emitted. Exposure from alpha radiation [with high linear energy transfer (LET)] is more damaging than low LET gamma rays and beta radiation per unit of absorbed dose. Radiation weighting factors are used to tackle this issue (supplementary Table S3). As effects of exposure to ionising radiation are additive, a thorough dose assessment should consider all exposure pathways for all radionuclides present, including any radioactive daughter products.

Here, the basic equations utilize radionuclide activity concentration data in soil and birds in order to estimate the external and internal absorbed dose rates (in μGy h^−1^) by birds of a given species, along with radionuclide-, species- and irradiation pathway-specific dose conversion coefficients (DCCs). The latter were determined per radionuclide and daughter(s) for adult stage of each species with the EDEN v3.1 software^35^.

The dose rate due to internal irradiation absorbed by the bird species *j* due to internalization of the radionuclide *r* (*IDR(j,r)* expressed in μGy h^−1^) is calculated according to equation (1):


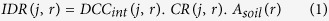


where

-*DCC*_*int*_*(j,r)* is the radionuclide-specific dose conversion coefficient for internal exposure of the species *j*, defined as the ratio between the dose rate to the bird species *j* and the activity concentration of radionuclide *r* in this bird (μGy h^−1^ per Bq kg^−1^ fresh weight);

-*CR(j,r)* is the concentration ratio of the radionuclide *r* by the bird species *j* and corresponds to the ratio between the activity concentration of the radionuclide in the bird species at the whole body level and the activity concentration of the same radionuclide in soil (kg dry weight soil/ kg fresh weight bird); this CR is known to be sensitive to the diet of the bird^22^;

-*A*_*soil*_*(r)* is _*the*_ activity of the radionuclide *r* in soil (Bq kg^−1^ of soil dry weight).

The dose rate due to external irradiation absorbed by the bird species *j* due to the presence of the radionuclide *r* in its neighborhood *(EDR(j,r)* expressed in μGy h^−1^) is calculated according to equation (2):


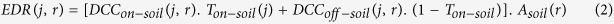


where

-*DCC*_*on-soil*_*(j,r)* is the dose conversion coefficient for the species *j* externally exposed when located on soil, to the radionuclide *r* present in soil (μGy h^−1^ per Bq kg^−1^ of soil);

-*T*_*on-soil*_*(j)* is the fraction of the time that the species *j* spends on soil (dimensionless);

-*DCC*_*off-soil*_*(j,r)* is the dose conversion coefficient for the species *j* externally exposed when located at a given distance from the soil surface to the radionuclide *r* present in soil (μGy h^−1^ per Bq kg^−1^ of soil).

Finally, the total dose rate absorbed by the bird species *j* exposed to all the radionuclides of interest is the sum of equations (1) and (2) applied for all radionuclides:


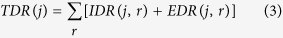


To estimate the total dose to birds, we assumed all species to be exposed to ionising radiation from the day of the accident (11 March 2011). Therefore, the total dose absorbed by each species *j* at a given site *i* calculated at the census date *k* noted *TD*_*ijk*_ was obtained according to:


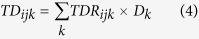


where *TDR*_*ijk*_ is the dose rate for species *j* at site *i* and census date *k*, and *D*_*k*_ is the duration of the period between the census date and the accident for year 2011 and the period between two successive census (ca. 365 days) for year 2012, 2013 and 2014.

Therefore we used the maximum estimates of the total dose for individuals yet hatched in 2011 when the accident occurred, resulting from an exposure duration of 112, 478, 843 and 1208 days for 2011, 2012, 2013 and 2014 census respectively.

### Bird species characteristics and assumptions needed for dose calculation

For each of the 57 species, we hypothesize that the individuals were present at the census point for breeding at the moment of the accident. We acknowledge that whatever the species, this assumption leads to a fairly realistic dose estimate for resident species, and to an overestimation that we consider to be the highest for migratory species exhibiting the shortest period of residence in the area. We assume the dose absorbed by individuals at the adult stage to be a reasonable proxy to represent the intensity of the stress potentially associated with the exposure to ionising radiation for the species, even though stages other than adults are ignored. For dose calculation for adults, a simplified representation of the body as ellipsoids of known mass and size (geometric characteristics) was adopted (supplementary Table S1), along with the media elementary composition (supplementary Table S2). DCCs were calculated on this basis for ^134^Cs, ^137^Cs and ^131^I (supplementary Table S3). In addition, each species was qualified by a simplified diet, either herbivorous or omnivorous to adopt the fit-for-purpose values of concentration ratios to quantify radionuclide aggregated transfer from soil to bird (supplementary Table S4). Finally, a bird’s life style was simplified by allocating to each species the associated fraction of time spent at a defined distance from soil, according to their mode of life (supplementary Table S1).

### Soil radioactive contamination data

The GPS coordinates defining each census point were used as input data to select the corresponding concentrations of radionuclides in soils on the basis of the monitoring performed by Japanese universities June-July 2011^20^. During these surveys, activities of ^134^Cs, ^137^Cs and ^131^I in samples of 5-cm layer bare soils were measured systematically at more than 2,000 sampling stations mainly located in the 80-km region from the Fukushima Daiichi NPP. The soil activity at the census point was assumed to be equal to the one measured at the nearest soil sampling point. Activity expressed in Bq m^−2^ was transformed into Bq kg^−1^ considering a conversion factor of 65 kg m^−2^ (sampling of a 5-cm soil layer with a density of 1300 kg m^−3^ in MEXT^36^). Several neighbor census points were associated with the same measurement point as shown in Fig. 1. The distances between bird census points and soil sampling points varied from 12 m to 1.6 km for cesium isotopes (and from 32 m to 3.3 km for iodine) and were ca. of the same order of magnitude as the spatial resolution of soil sampling (systematic grid of 2×2 km^2^). Radioactive decay was considered calculating the time elapsed between the accident date and the census date, with a physical decay of 2.2 y, 30.2 y and 8 d for ^134^Cs, ^137^Cs and ^131^I respectively. Considering only those three radioisotopes was of very small influence in our dose estimates. On the basis of maximum soil deposition concentrations for ^134^Cs, ^137^Cs, ^131^I, ^129m^Te, ^110m^Ag, ^89^Sr, ^90^Sr, ^238^Pu, ^240^Pu reported by Saito *et al.*^20^, we estimated the two cesium isotopes contributed to 78 to 97% of the total dose rate estimates for the two species characterized by the minimum (*Anas poecilorhyncha*) and maximum (*Phasianus colchicus*) absorbed dose rate in 2011, and that their contribution tended to 100% from 2012 onwards. At the first census, four months after the accident, ^131^I only accounted for a maximum of 0.15% of the total dose rates of birds due to the short half-life of this isotope (8.02 days).

### Statistics

The total doses (*TD*_*ijk*_), reconstructed per species *j* per site *i* and census year *k*, were weighted by the observed relative species abundances (*A*_*ijk*_*/TNB*_*ik*_) and then summed for all the species in order to obtain a unique value (*TD*_*ik*_) per site and per year as follows:


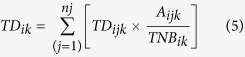


where *A*_*ijk*_ is the abundance of species *j* observed at a site *i* during year *k*; *TNB*_*ik*_ is the total number of birds observed at a census site *i* for year *k*, corresponding to the sum of all individuals of all species at site *i* during census year *k* and *nj* is the number of species in the whole study.

This weighted average total dose is a representative indicator of the radiological stress intensity at a site *i* during year *k*.

Simpson’s index of diversity^37^ for a site *i* at a year *k* is defined as:


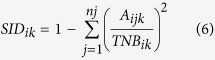


This index represents the probability that two individuals randomly observed in a census site *i* of year *k* will belong to different species.

Generalized Linear Mixed Models (GLMM), assuming Poisson error distribution, and Linear Mixed Models (LMM) were used to assess the effect of total dose on total number of birds and Simpson’s index of diversity, respectively. After checking for multicollinearity (i.e. omitting variables with Pearson correlation coefficient ≥0.87; supplementary Table S5), the possible confounding variables included in models were time, squared time (because bird activity can be curvilinear with time), cloud cover, temperature, wind (as a categorial variable), altitude, %Grass, %Farmland, and %Coniferous. The main predictor, i.e. total dose was logarithm (base 10) transformed in order to improve linearity with total number of birds and Simpson’s index of diversity. To facilitate interpretation of regression coefficients, inputs variables were standardized by dividing by two standard deviations^38^,^39^.

Site and the combination of census day and transect were included as random effects in the GLMM, while site, census day and transect were considered in the LMM. These specific random structures were selected after comparing several models, using the Akaike Information Criterion (AIC) (supplementary Table S6). LMMs were fitted using restricted maximum likelihood estimation; GLMMs were fitted using maximum likelihood estimation. Effects of predictors on total number of birds and Simpson’s index of diversity were assessed using model-based approximate Wald z-statistic test and t test with Satterthwaite’s approximation to degrees of freedom^40^. The significance level was fixed at 5%. All analyses were performed with the R software, version 3.1.2^41^ and RStudio environment version 0.98.1091^42^. GLMM and LMM were fitted using the package *lme4*^43^.

Since the effect of total dose was assessed as significant on total number of birds, we estimated the level giving 50% loss in total abundance (i.e. the ED_50_ critical radiotoxicity endpoint). From the fitted GLMM the dose-response relationship between total dose and total number of birds was predicted for a subset of doses, which were chosen in such a way that they were uniformly distributed over the range of calculated doses. Predicted values for total number of birds were augmented with random errors to recover the variation in the original data. Such predicted dose-response curves were generated many times. For each curve, ED_50_ was subsequently estimated using a constrained log-logistic model and these estimates were summarized to give the final estimate. The details of the algorithm used for this approach is described in supplementary Table S7.

## Additional Information

**How to cite this article**: Garnier-Laplace, J. *et al.* Radiological dose reconstruction for birds reconciles outcomes of Fukushima with knowledge of dose-effects relationships. *Sci. Rep.* **5**, 16594; doi: 10.1038/srep16594 (2015).

## Supplementary Material

Supporting Information

## Figures and Tables

**Table 1:** Range of variation in measured ambient radiation among the 300 census sites per year and corresponding range of variation among the 57 species of calculated dose rates for adult birds.

Year	Ambient radiation range among sites (μGy/h^-1^)	Absorbed dose rates among sites and among species (μGy/h^-1^)
2011	0.55–31	0.28–97
2012	0.21–28	0.24–84
2013	0.18–21	0.21–74
2014	0.16–13	0.19–66

All values are rounded to 2 significant digits.

**Table 2:** Total number of birds: effects of log_10_ total dose and confounding variables.

Random effects:				
Groups	Variance	SD		
Site	0.052	0.228		
CensDayTra^a^	0.023	0.150		
Fixed effects:				
	Estimate	SE	z	p
(Intercept)	1.666	0.048	34.650	<0.0001
** log**_**10**_ **Total dose**	**−0.299**	**0.074**	**−4.060**	**<0.0001**
	**(−0.256)**^b^	**(0.063)**^b^		
Time	**−**0.054	0.047	**−**1.160	0.246
** Time^2^**	**0.352**	**0.070**	**5.050**	**<0.0001**
Cloud cover	0.016	0.053	0.290	0.769
** Temperature**	**−0.182**	**0.076**	**−2.400**	**0.016**
Wind 1^c^	0.079	0.096	0.820	0.410
Wind 2	**−**0.041	0.068	**−**0.600	0.549
** Wind 3**	**−0.290**	**0.101**	**−2.880**	**0.004**
** Wind 4**	**−0.471**	**0.170**	**−2.770**	**0.006**
Wind 5	**−**0.020	0.139	**−**0.140	0.886
** Altitude**	**−0.275**	**0.046**	**−5.980**	**<0.0001**
** Grass**	**−0.173**	**0.035**	**−4.980**	**<0.0001**
** Farmland**	**0.266**	**0.046**	**5.790**	**<0.0001**
Coniferous	0.011	0.015	0.720	0.470

Effect size was standardised by two SD. Parameters with a significant effect (p < 0.05) are highlighted in bold.

^a^CensDayTra is the combination of Census day and Transect categories.

^b^Unstandardised partial slope values obtained after model fit with the same fixed and random effects, but without standardization of the input variables.

^c^Wind 0 was the reference category.

**Table 3:** Simpson’s index of diversity: effects of log_10_ total dose and confounding variables.

Random effects:					
Groups	Variance	SD			
Site	0.002	0.042			
Census Day	0.001	0.028			
Transect	0.004	0.063			
Residual	0.026	0.160			
Fixed effects:					
	Estimate	SE	df	t	p
(Intercept)	0.687	0.035	3.40	19.846	<0.001
** Total Dose Log**	**0.053**	**0.023**	**55.30**	**2.302**	**0.025**
	**(0.045)**^b^	**(0.020)**^b^			
** Time**	**−0.084**	**0.015**	**145.90**	**−5.643**	**<0.0001**
Time^2^	0.008	0.023	974.40	0.323	0.747
Cloud cover	0.006	0.015	123.20	0.389	0.698
Temp	−0.001	0.019	18.20	−0.033	0.974
Wind1^a^	0.009	0.026	48.10	0.335	0.739
Wind2	0.007	0.023	26.80	0.306	0.762
** Wind3**	**−0.150**	**0.031**	**309.90**	**−4.806**	**<0.0001**
** Wind4**	**−0.125**	**0.043**	**20.90**	**−2.901**	**0.009**
Wind5	−0.026	0.037	19.20	−0.682	0.503
** Altitude**	**−0.040**	**0.014**	**318.90**	**−2.888**	**0.004**
Grass	0.005	0.011	292.60	0.456	0.649
** Farmland**	**0.089**	**0.014**	**299.20**	**6.594**	**<0.0001**
Coniferous	0.010	0.011	282.60	0.928	0.354

Effect size was standardised by two SD. Parameters with a significant effect (p < 0.05) are highlighted in bold.

^a^Wind 0 was the reference category.

^b^Unstandardised partial slope values obtained after model fit with the same fixed and random effects, but without standardization of the input variables.
