# Supplementary material for: Radiological dose reconstruction for birds reconciles outcomes of Fukushima with knowledge of dose-effect relationships
Source: Sci Rep. 2015 Nov 16;5:16594. doi: 10.1038/srep16594 (PMC4645120; doi:10.1038/srep16594)
Supplement: Supporting Information [file srep16594-s1.pdf]

Radiological dose reconstruction for birds reconciles outcomes of Fukushima with knowledge of dose-effect relationships

by Jacqueline Garnier-Laplace, Karine Beaugelin-Seiller, Claire Della-Vedova, Jean-Michel Métivier, Timothy A. Mousseau, and Anders Pape Møller

Supporting Information

Table S1. Bird species characteristics needed for radiological dose calculations (see equation (2) in the main text)

					Size data**	
					Long axis	Short axis
Latin name	Diet*	Distance to soil (m)	Time on soil	Body mass (g)	(cm)	(cm)
Accipiter gentilis	1	5.00	0.20	1024.5	35.5	7.2
Acrocephalus arundinaceus	1	0.50	1.00	30.35	12.3	2.1
Aegithalos caudatus	1	3.00	0.10	8.8	5.0	1.8
Alauda arvensis	1	0.00	1.00	36.4	11.6	2.4
Alcedo atthis	1	1.00	1.00	35.8	15.7	2.0
Anas poecilorhyncha	0	0.00	1.00	1220	48.6	6.7
Apus affinis	1	10.00	0.00	17.9	6.9	2.2
Ardea cinerea	1	0.00	1.00	1443	75.0	5.9
Bambusicola thoracica	0	0.00	1.00	270.25	15.8	5.5
Butastur indicus	1	3.00	0.30	397	23.7	5.5
Buteo buteo	1	3.00	0.30	806.5	36.5	6.3
Carduelis sinica	0	5.00	0.20	31.3	8.7	2.5
Cettia diphone	1	1.00	0.05	14	9.6	1.6
Cinclus pallasii	1	0.00	1.00	76	14.4	3.1
Cisticola juncidis	1	0.00	1.00	8.5	6.0	1.6
Corvus corone	1	2.00	0.50	544.5	27.3	6.0
Corvus macrorhynchos	1	2.00	0.50	483	34.0	5.0
Cuculus canorus	1	5.00	0.10	113	19.0	3.3
Cuculus poliocephalus	1	5.00	0.10	41	16.0	2.1
Cuculus saturatus	1	5.00	0.10	96.5	17.5	3.1
Cyanopica cyanus	1	5.00	0.10	71	18.0	2.7

Latin name	Diet*	Distance to soil (m)	Time on soil	Body mass (g)	Size data** Long axis (cm)	Size data** Short axis (cm)
Cyanoptila cyanomelana	1	5.00	0.05	25	9.5	2.2
Delichon urbica	1	5.00	0.10	19.55	6.9	2.2
Dendrocopos kizuki	1	3.00	0.00	21.8	9.9	2.0
Emberiza cioides	0	1.00	0.90	21	9.3	2.0
Eophona personata	0	5.00	0.00	80	11.6	3.5
Falco peregrinus	1	5.00	0.10	889.25	34.5	6.8
Falco tinnunculus	1	3.00	0.10	201.5	18.0	4.5
Ficedula narcissina	1	5.00	0.05	14	6.0	2.0
Garrulax garrulax	1	5.00	0.00	116	17.0	3.5
Garrulus glandarius	0	5.00	0.10	161.7	21.0	3.7
Hirundo rustica	1	3.00	0.10	19.1	9.5	1.9
Hypsipetes amaurotis	1	5.00	0.05	85	16.5	3.0
Lanius bucephalus	1	3.00	0.50	41.75	11.3	2.6
Milvus migrans	1	3.00	0.20	828.5	32.0	6.8
Motacilla alba	1	0.00	1.00	20.75	9.6	2.0
Motacilla cinerea	1	0.00	1.00	17.35	8.3	1.9
Motacilla grandis	1	0.00	1.00	33	15.3	2.0
Parus ater	1	5.00	0.05	9.25	6.4	1.6
Parus major	1	5.00	0.05	18.5	7.9	2.0
Parus montanus	1	5.00	0.05	11.65	5.3	2.0
Parus varius	1	5.00	0.05	17	8.8	1.9
Passer montanus	0	1.00	0.80	21.7	8.8	2.1
Pericrocotus divaricatus	1	5.00	0.00	21.6	8.3	2.2
Phalacrocorax carbo	1	0.00	1.00	2110	76.8	7.0
Phasianus colchicus	0	0.00	1.00	1400	22.8	10.5
Phasianus soemmeringii	0	0.00	1.00	907	27.7	7.6
Picus awokera	1	5.00	0.50	129	***19.1***	3.5
Regulus regulus	1	5.00	0.00	5.7	4.9	1.4
Streptopelia orientalis	0	3.00	0.50	214.5	20.8	4.3
Sturnus cineraceus	1	1.00	0.50	62	17.7	2.5

Latin name	Diet*	Distance to soil (m)	Time on soil	Body mass (g)	Size data** Long axis (cm)	Short axis (cm)
Tarsiger cyanurus	1	3.00	0.50	12.1	8.3	1.6
Terpsiphone atrocaudata	1	5.00	0.00	18.7	18.0	1.4
Troglodytes troglodytes	1	5.00	0.20	8.9	6.9	1.5
Turdus cardis	1	3.00	0.50	57.5	14.6	2.7
Urosphaera squameiceps	1	1.00	0.10	9.5	7.6	1.5
Zosterops japonica	1	5.00	0.00	10	6.6	1.6

*0: herbivorous / 1: omnivorous; ** based on an equivalent revolution ellipsoid; size estimated from mass and body length, considering a density of 1.07, and a shape defined by a revolution ellipsoid (two axes to determine)

Table S2. Media elementary composition needed for DCCs calculation *via* the EDEN v3 software (Beaugelin-Seiller et al., 2006)

	Density (g/cm^3)	Mass composition (%) Al	Ar	C	Ca	Fe	H	K	Mg	N	Na	O	P	S	Si
soil	$1.7\ 10^{0}$	$7.4\ 10^{0}$	n.d.	$1.8\ 10^{-1}$	$3.3\ 10^{0}$	$4.2\ 10^{0}$	$9.6\ 10^{-1}$	$2.3\ 10^{0}$	$2.1\ 10^{0}$	$3.0\ 10^{-2}$	$2.4\ 10^{0}$	$5.1\ 10^{1}$	$1.1\ 10^{-1}$	$1.1\ 10^{-1}$	$2.6\ 10^{1}$
air	$1.3\ 10^{-3}$	n.d.	$1.3\ 10^{0}$	$1.0\ 10^{-2}$	n.d.	n.d.	$6.0\ 10^{-2}$	n.d.	n.d.	$7.5\ 10^{1}$	n.d.	$2.4\ 10^{1}$	n.d.	n.d.	n.d.
animal	$1.1\ 10^{0}$	n.d.	n.d.	$1.9\ 10^{1}$	$1.4\ 10^{0}$	$1.0\ 10^{-2}$	$9.3\ 10^{0}$	$2.2\ 10^{-1}$	$4.0\ 10^{-2}$	$5.2\ 10^{0}$	$2.6\ 10^{-1}$	$6.3\ 10^{1}$	$6.3\ 10^{-1}$	$6.4\ 10^{-1}$	n.d.

Table S3. DCC estimates (Gy.d^{-1} per Bq.kg^{-1}) per radionuclide, irradiation pathways and species (adult stage only), weighted assuming factors of 10 for α-radiation, 3 for low-β radiation (E < 10 keV), and 1 for other β-radiation and γ-radiation. The characteristics of the exposure scenes used for DCC calculation are as follows: for birds allocating their time-budget between soil and air: adult bird on soil, and adult bird in air at a given distance to soil as indicated in Table S1, with an occupancy factor for each scene (always the long axis parallel to the soil, to maximize the exposure); some birds live always on soil (time on soil=1 in Table S1), some others never come on soil (time on soil=0 in Table S1). Use 1 Gy d^{-1} = 4.17 x 10^{4} µGy h^{-1} in case conversion is needed.

	internal exposure			external exposure to soil - contact			external exposure to soil - distance		
	^{137}Cs+	^{134}Cs	^{131}I	^{137}Cs+	^{134}Cs	^{131}I	^{137}Cs+	^{134}Cs	^{131}I
Accipiter gentilis	9.54E-09	6.65E-09	7.07E-09	2.04E-09	2.42E-09	2.10E-09	6.70E-10	1.91E-09	3.09E-10
Acrocephalus arundinaceus	9.54E-09	6.65E-09	7.07E-09	7.72E-11	2.18E-10	4.04E-11	7.33E-10	2.37E-09	3.76E-10
Aegithalos caudatus	9.43E-09	6.49E-09	7.01E-09	7.96E-11	2.22E-10	4.32E-11	9.75E-10	2.77E-09	4.88E-10
Alauda arvensis	9.61E-09	6.76E-09	7.11E-09	6.49E-10	6.21E-10	5.87E-10	No exposure at distance		
Alcedo atthis	9.51E-09	6.63E-09	7.06E-09	7.55E-11	2.12E-10	3.97E-11	8.11E-10	2.50E-09	4.27E-10
Anas poecilorhyncha	1.03E-08	8.21E-09	7.53E-09	3.17E-10	5.47E-10	2.59E-10	No exposure at distance		
Apus affinis	9.54E-09	6.65E-09	7.08E-09	1.20E-09	1.03E-09	1.12E-09	8.82E-10	2.48E-09	4.50E-10
Ardea cinerea	1.02E-08	7.99E-09	7.47E-09	2.81E-10	4.96E-10	2.27E-10	No exposure at distance		
Bambusicola thoracica	1.01E-08	7.71E-09	7.40E-09	5.65E-10	7.92E-10	5.46E-10	No exposure at distance		
Butastur indicus	1.01E-08	7.78E-09	7.42E-09	4.71E-10	6.69E-10	4.48E-10	7.40E-10	2.12E-09	3.49E-10
Buteo buteo	1.02E-08	8.06E-09	7.49E-09	3.38E-10	5.84E-10	2.77E-10	6.92E-10	1.99E-09	3.22E-10
Carduelis sinica	9.61E-09	6.77E-09	7.12E-09	7.47E-10	6.90E-10	6.83E-10	9.37E-10	2.64E-09	4.62E-10
Cettia diphone	9.40E-09	6.45E-09	6.99E-09	7.57E-11	2.11E-10	4.10E-11	8.39E-10	2.58E-09	4.44E-10
Cinclus pallasii	9.74E-09	7.01E-09	7.19E-09	4.44E-10	4.83E-10	3.89E-10	No exposure at distance		
Cisticola juncidis	9.38E-09	6.43E-09	6.99E-09	7.76E-11	2.15E-10	4.28E-11	No exposure at distance		
Corvus corone	1.02E-08	7.94E-09	7.46E-09	4.41E-10	6.62E-10	4.08E-10	6.76E-10	1.98E-09	3.16E-10
Corvus macrorhynchos	1.00E-08	7.66E-09	7.38E-09	4.38E-10	6.41E-10	4.08E-10	7.20E-10	2.10E-09	3.40E-10
Cuculus canorus	9.78E-09	7.09E-09	7.22E-09	4.28E-10	4.83E-10	3.70E-10	8.77E-10	2.48E-09	4.26E-10
Cuculus poliocephalus	9.54E-09	6.66E-09	7.08E-09	7.34E-11	2.06E-10	3.87E-11	9.56E-10	2.69E-09	4.71E-10
Cuculus saturatus	9.61E-09	6.77E-09	7.12E-09	7.47E-10	6.90E-10	6.83E-10	9.37E-10	2.64E-09	4.62E-10
Cyanopica cyanus	9.68E-09	6.89E-09	7.15E-09	5.80E-10	5.75E-10	5.19E-10	9.14E-10	2.58E-09	4.48E-10
Cyanoptila cyanomelana	9.56E-09	6.68E-09	7.08E-09	7.65E-11	2.16E-10	4.01E-11	9.56E-10	2.68E-09	4.71E-10
Delichon urbica	9.54E-09	6.65E-09	7.08E-09	3.64E-10	4.16E-10	3.17E-10	9.56E-10	2.70E-09	4.75E-10
Dendrocopos kizuki	9.50E-09	6.60E-09	7.06E-09	7.65E-11	2.15E-10	4.04E-11	9.56E-10	2.70E-09	4.73E-10
Emberiza cioides	9.50E-09	6.60E-09	7.06E-09	7.68E-11	2.17E-10	4.06E-11	8.16E-10	2.51E-09	4.30E-10
Eophona personata	9.80E-09	7.11E-09	7.23E-09	2.90E-10	3.79E-10	2.39E-10	8.74E-10	2.47E-09	4.25E-10
Falco peregrinus	1.03E-08	8.20E-09	7.53E-09	3.16E-10	5.38E-10	2.62E-10	6.88E-10	1.96E-09	3.19E-10

	internal exposure			external exposure to soil - contact			external exposure to soil - distance		
	$^{137}Cs+$	^{134}Cs	^{131}I	$^{137}Cs+$	^{134}Cs	^{131}I	$^{137}Cs+$	^{134}Cs	^{131}I
Falco tinnunculus	9.81E-09	7.15E-09	7.24E-09	4.96E-10	7.38E-10	4.57E-10	8.50E-10	2.42E-09	4.13E-10
Ficedula narcissina	9.49E-09	6.57E-09	7.05E-09	7.68E-11	2.16E-10	4.13E-11	9.75E-10	2.74E-09	4.84E-10
Garrulax garrulax	9.81E-09	7.15E-09	7.24E-09	3.31E-10	4.14E-10	2.76E-10	8.67E-10	2.45E-09	4.20E-10
Garrulus glandarius	9.85E-09	7.23E-09	7.26E-09	3.28E-10	4.42E-10	2.65E-10	8.52E-10	2.41E-09	4.12E-10
Hirundo rustica	9.47E-09	6.56E-09	7.04E-09	7.74E-11	2.18E-10	4.10E-11	9.56E-10	2.72E-09	4.77E-10
Hypsipetes amaurotis	9.72E-09	6.98E-09	7.19E-09	3.92E-10	4.55E-10	3.37E-10	8.97E-10	2.53E-09	4.38E-10
Lanius bucephalus	9.65E-09	6.82E-09	7.13E-09	1.97E-10	4.57E-10	1.10E-10	9.13E-10	2.59E-09	4.49E-10
Milvus migrans	1.03E-08	8.19E-09	7.53E-09	3.13E-10	5.71E-10	2.44E-10	6.72E-10	1.94E-09	3.11E-10
Motacilla alba	9.50E-09	6.61E-09	7.06E-09	7.43E-11	2.09E-10	3.94E-11	No exposure at distance		
Motacilla cinerea	9.47E-09	6.56E-09	7.04E-09	7.48E-11	2.11E-10	4.00E-11	No exposure at distance		
Motacilla grandis	9.51E-09	6.63E-09	7.06E-09	7.70E-11	2.16E-10	4.04E-11	No exposure at distance		
Parus ater	9.38E-09	6.43E-09	6.99E-09	7.79E-11	2.18E-10	4.24E-11	9.94E-10	2.80E-09	4.97E-10
Parus major	9.50E-09	6.59E-09	7.06E-09	7.79E-11	2.20E-10	4.12E-11	9.66E-10	2.72E-09	4.80E-10
Parus montanus	9.48E-09	6.56E-09	7.04E-09	7.75E-11	2.18E-10	4.17E-11	9.75E-10	2.74E-09	4.85E-10
Parus varius	9.47E-09	6.56E-09	7.04E-09	7.67E-11	2.16E-10	4.08E-11	9.75E-10	2.74E-09	4.83E-10
Passer montanus	9.53E-09	6.64E-09	7.07E-09	7.64E-11	2.16E-10	4.03E-11	8.13E-10	2.50E-09	4.27E-10
Pericrocotus divaricatus	9.55E-09	6.67E-09	7.08E-09	1.13E-09	9.46E-10	1.06E-09	9.56E-10	2.69E-09	4.73E-10
Phalacrocorax carbo	1.03E-08	8.32E-09	7.56E-09	2.83E-10	5.42E-10	2.09E-10	No exposure at distance		
Phasianus colchicus	1.06E-08	9.04E-09	7.76E-09	2.47E-10	3.43E-10	6.09E-11	No exposure at distance		
Phasianus soemmeringii	1.03E-08	8.38E-09	7.58E-09	2.76E-10	3.81E-10	7.78E-11	No exposure at distance		
Picus awokera	9.82E-09	7.16E-09	7.24E-09	1.92E-10	3.25E-10	1.40E-10	8.65E-10	2.44E-09	4.19E-10
Regulus regulus	9.30E-09	6.34E-09	6.95E-09	7.87E-11	2.18E-10	4.35E-11	1.01E-09	2.84E-09	5.06E-10
Streptopelia orientalis	9.93E-09	7.41E-09	7.31E-09	3.72E-10	5.61E-10	3.38E-10	8.03E-10	2.30E-09	3.85E-10
Sturnus cineraceus	9.63E-09	6.82E-09	7.13E-09	7.29E-10	6.70E-10	6.66E-10	7.80E-10	2.41E-09	4.07E-10
Tarsiger cyanurus	9.39E-09	6.44E-09	6.99E-09	7.64E-11	2.13E-10	4.14E-11	9.85E-10	2.78E-09	4.90E-10
Terpsiphone atrocaudata	9.33E-09	6.37E-09	6.96E-09	1.09E-09	9.13E-10	1.02E-09	1.00E-09	2.81E-09	5.00E-10
Troglodytes troglodytes	9.35E-09	6.40E-09	6.97E-09	7.75E-11	2.16E-10	4.24E-11	1.00E-09	2.81E-09	5.00E-10
Turdus cardis	9.67E-09	6.88E-09	7.15E-09	5.28E-10	5.38E-10	4.71E-10	9.02E-10	2.57E-09	4.43E-10
Urosphaera squameiceps	9.35E-09	6.40E-09	6.97E-09	7.70E-11	2.14E-10	4.20E-11	8.48E-10	2.60E-09	4.49E-10
Zosterops japonica	9.38E-09	6.43E-09	6.99E-09	7.74E-11	2.15E-10	4.24E-11	9.94E-10	2.80E-09	4.97E-10

Table S4. Aggregated Concentration Ratios (Bq/kg w.w. whole body per Bq/kg d.w soil) for cesium and iodine. For cesium, the value was attributed to bird species according to their diet, except for a limited number of species where species-specific data were available or when the species-specific data could be extrapolated from a species of the "same genus and diet" for which CR values exist. For iodine the same value was applied to all species since there is no specific data in the literature except the value proposed in the ERICA database for vertebrates.

Species or group of species	Extrapolation rules	**Aggregated CR**	Type of values and number of data	Reference
**Cesium**				
**Herbivorous birds**[1]	Same diet	1	Arithmetic mean of 57 data	IAEA (2014)[3]
**Omnivorous birds**[2]	Same diet	0.57	Arithmetic mean of 79 data	IAEA (2014)
Anas poecilorhyncha	Same genus and diet as *Anas platyrhynchos*	0.095	Arithmetic mean of 2 data	Extracted from the WTD[4]
Accipiter gentilis	Same species	0.0926	1 datum	Extracted from the WTD
Hirundo rustica	Same species	0.081	1 datum	Extracted from the WTD
Lanius bucephalus	Same diet and genus as *Lanius collurio*	0.488	Arithmetic mean of 2 data	Extracted from the WTD
Parus ater, Parus montanus, Parus varius	Same diet and genus as *Parus major*	0.559		
Parus major	Same species	0.559	Arithmetic mean of 26 data	Extracted from the WTD
Turdus cardis	Same diet and genus as *Turdus pilaris*	0.0517	Arithmetic mean of 3 data	Extracted from the WTD
Sturnus cineraceus	Same diet and genus as *Sturnus vulgaris*	0.174	1 datum	Extracted from the WTD
**Iodine**				
All species	Same as vertebrates (a model derived value for mammals)	0.4	No information	ERICA tool v1.2, Nov 2014 www.erica-tool.com

[1] *Bambusicola thoracica, Carduelis sinica, Emberiza cioides, Eophona personata, Garrulus glandarius, Phasianus colchicus, Phasianus soemmeringii, Streptopelia orientalis*

[2]*Accipiter gentilis, Acrocephalus arundinaceus, Aegithalos caudatus, Alauda arvensis, Alcedo atthis, Apus affinis, Ardea cinerea, Butastur indicus, Buteo buteo, Cettia diphone, Cinclus pallasii, Cisticola juncidis, Corvus corone, Corvus macrorhynchos, Cuculus canorus, Cuculus poliocephalus, Cuculus saturates, Cyanopica cyanus, Cyanoptila cyanomelana, Delichon urbica, Dendrocopos kizuki, Falco peregrinus, Falco tinnunculus, Ficedula narcissina, Garrulax garrulax, Hypsipetes amaurotis, Milvus migrans, Motacilla alba, Motacilla cinerea, Motacilla grandis, , Passer montanus, Pericrocotus divaricatus, Phalacrocorax carbo, Picus awokera, Regulus regulus, Tarsiger cyanurus, Terpsiphone atrocaudata, Troglodytes troglodytes, Urosphaera squameiceps, Zosterops japonica*

[3]IAEA (2014). Handbook of parameter values for the prediction of radionuclide tranfers to wildlife. Technical Report Series n°479, Vienna, 218 pages.

[4]Extracted from the wildlife transfer database (WTD; www.wildlifetransferdatabase.org) version as described by Copplestone, D., Beresford, N.A., Brown, J., Yankovich, T. 2013. An international database of radionuclide Concentration Ratios for wildlife: development and uses. J. Environ. Radioact. 126, 288-298. http://dx.doi.org/10.1016/j.jenvrad.2013.05.007

Table S5. Pearson correlation matrix of raw possible explanatory variables. Pearson correlation coefficient ≥ |0.85| are highlighted in bold.

	Total dose	Time	Cloud cover	Temperature	Wind	Altitude	Grass	Bush	Tree	Farmland	Deciduous	Coniferous
Total dose	1.00											
Time	-0.09	1.00										
Cloud cover	0.19	0.31	1.00									
Temp	-0.28	0.38	-0.03	1.00								
Wind	-0.15	0.00	0.08	-0.06	1.00							
Altitude	0.13	0.17	0.13	0.09	-0.23	1.00						
Grass	-0.03	0.04	0.04	0.00	-0.07	0.22	1.00					
Bush	0.40	-0.08	0.07	-0.01	-0.07	0.28	0.09	1.00				
Tree	0.43	-0.04	0.08	-0.01	-0.13	0.35	0.10	**0.92**	1.00			
Farmland	-0.40	0.11	-0.03	0.01	0.05	-0.22	0.11	**-0.90**	**-0.87**	1.00		
Deciduous	0.43	-0.04	0.08	-0.01	-0.13	0.34	0.09	**0.92**	1.00	**-0.87**	1.00	
Coniferous	0.02	0.00	0.00	0.02	0.01	0.10	0.03	0.10	0.13	-0.10	0.07	1.00

Table S6. AIC of GLMM (used to study Total number of birds) and LMM (used to study Simpson's index of diversity) containing different random effect structures. Fixed effects are the same (i.e log_{10} Total dose, Time, Time², Cloud cover, Temperature, Wind, Altitude, Grass, Farmland, Coniferous). CensDay is the census day (i.e combination of Year and Date factors). CensDayTra is the combination of modalities of Census Day and Transect factors. The lowest AIC are highlighted in bold.

	AIC	
Random effect structure	Total Number of Birds	Simpson's Index of Diversity
no random effect	5763.43	-732.18
1|Year	5958.63	-728.29
1|Site	5772.62	-737.83
1|Transect	5945.18	-759.28
1|CensDay	5924.98	-736.70
1|CensDayTra	5898.54	-754.34
1|Year + 1|Site	5766.86	-736.10
1|Year + 1|Transect	5939.85	-762.28
1|Site + 1|Transect	5770.70	-762.52
1|Site + 1|CensDay	5734.57	-743.79
1|Site + 1|CensDayTra	**5728.78**	-759.15
1|Transect + 1|CensDay	5906.67	-764.18
1|Site + 1|Transect + 1|CensDay	5732.89	**-768.61**

Table S7. Algorithm used to estimate ED_{50} from the fitted GLMM dose-response relationship between total dose (TD) and total number of birds (TNB).

A. Split the set of calculated Total Dose into 9 doses interval, created by geometric progression from min (Total Dose), to max (Total Dose). Let I_i denote the ith interval

B. For i=1 to 9 :

   1. Randomly select 5 Total Doses into I_i. Let TD_{ij} denotes the jth selected dose of the ith interval.

      a. For j = 1 to 5 :

         i) use *predictSE* function implemented in *AICcmodavg* package[S1] to estimate an ‘average’ TNB, denoted A_TNB_{ij} and it’s standard error, denoted SE_TNB_{ij}

         ii) generate a ‘random’ TNB, denoted $R\_TNB\__{ij}$, from Gaussian distribution $\mathcal{N}(A\_TNB_{ij}, SE\_TNB_{ij})$

C. Using *drm* function of *drc* package[S2], fit a five-parameter log-logistic model to the (TD_{ij}; R_TNB_{ij}) data, forcing to pass by the pair (TD=0.0012*; TNB=8), and fixing the highest and lowest limits of the curve, respectively to 8 and 0.

D. Estimate ED_{50} from the fit using *ED* function of *drc* package

E. Use bootstrap approach to estimate 95% confidence interval to the derived ED_{50} , redoing step B to D a large amount of times (here 4999) and using the 2.5^{th} and 97.5^{th} percentiles of the 4999 obtained values.

*0.0012 Gy is the background reference level reported for bird-type organism 0.01 mGy d^{-1} by ICRP 108[21] multiplied by 1208 days which is the maximum exposure duration of birds in this study.

[S1]. Mazerolle, M.J. AICcmodavg: Model selection and multimodel inference based on (Q)AIC(c). R package version 2.0-3. http://cran.r-project.org/web/packages/AICcmodavg/, Accessed 2 June, 2015.

[S2]. Ritz, C. & Streibig, J. C. Bioassay Analysis using R. J. Statist. Software, Vol 12, Issue 5. R package version 2.5-12. http://cran.r-project.org/web/packages/drc/ (2005)
